# Near-Infrared Autofluorescence: Early Detection of Retinal Pigment Epithelial Alterations in Inherited Retinal Dystrophies

**DOI:** 10.3390/jcm13226886

**Published:** 2024-11-15

**Authors:** Simone Kellner, Silke Weinitz, Ghazaleh Farmand, Ulrich Kellner

**Affiliations:** 1 Rare Retinal Disease Center, Augen Zentrum Siegburg, MVZ Augenärztliches Diagnostik- und Therapiecentrum Siegburg GmbH, 53721 Siegburg, Germany; s.kellner@osg.de (S.K.);; 2 RetinaScience, 53192 Bonn, Germany

**Keywords:** near-infrared autofluorescence, fundus autofluorescence, inherited chorioretinal dystrophies, Best disease, ABCA4-associated chorioretinal dystrophies, retinitis pigmentosa, cone-rod dystrophies

## Abstract

Near-infrared autofluorescence (NIA) is a non-invasive retinal imaging technique used to examine the retinal pigment epithelium (RPE) based on the autofluorescence of melanin. Melanin has several functions within RPE cells. It serves as a protective antioxidative factor and is involved in the phagocytosis of photoreceptor outer segments. Disorders affecting the photoreceptor–RPE complex result in alterations of RPE cells which are detectable by alterations of NIA. NIA allows us to detect early alterations in various chorioretinal disorders, frequently before they are ophthalmoscopically visible and often prior to alterations in lipofuscin-associated fundus autofluorescence (FAF) or optical coherence tomography (OCT). Although NIA and FAF relate to disorders affecting the RPE, the findings for both imaging methods differ and the area involved has been demonstrated to be larger in NIA compared to FAF in several disorders, especially inherited retinal dystrophies (IRDs), indicating that NIA detects earlier alterations compared to FAF. Foveal alterations can be much more easily detected using NIA compared to FAF. A reduced subfoveal NIA intensity is the earliest sign of autosomal dominant Best disease, when FAF and OCT are still normal. In other IRDs, a preserved subfoveal NIA intensity is associated with good visual acuity. So far, the current knowledge on NIA in IRD has been presented in multiple separate publications but has not been summarized in an overview. This review presents the current knowledge on NIA in IRD and demonstrates NIA biomarkers.

## 1. Introduction

Near-infrared autofluorescence (NIA) is a non-invasive imaging technique used to examine the retinal pigment epithelium that was introduced in 2006 by Keilhauer and Delori [1]. NIA is based on the autofluorescence of melanin excited with 787 nm (near-infrared) illumination, whereas fundus autofluorescence (FAF) is based on the autofluorescence of lipofuscin excited with 476–585 nm illumination [2,3,4].

Melanin is the predominant protective agent of RPE cells with antioxidative properties and light absorption potential, which allow them to reduce radiation damage and light scattering [5]. Melanin granules are located in the apical region of the RPE cells [6]. Melanin granules participate in the phagocytosis of photoreceptor outer segments and can develop into melanolipofuscin granules, which also contribute to NIA [7,8]. In addition, oxidation can influence melanin autofluorescence [9]. The concentration of melanin in RPE cells is highest under the fovea and declines toward the parafovea, but then remains constant towards the far periphery [10]. The concentration of melanin slowly declines over a lifetime. Melanogenesis occurs in adults, and can be increased during disease processes [11,12]. Only a limited amount of melanin is located in the choroid. Melanin emits autofluorescence; the highest amount of autofluorescence can be excited with 785 nm, close to the 787 nm used for NIA. Emitted light of ≥800 nm is measured to obtain NIA images.

Lipofuscin and melanin are the major fluorophores predominantly located within the RPE cells. Therefore, both NIA and FAF can map disease-associated alterations of the RPE cells at the entire fundus. Multiple other fluorophores exist in retina and RPE, which might make a small contribution to NIA and FAF. However, an in vivo examination technique has been described for only one other fluorophore: flavoprotein [13]. This is also due to the fact that NIA and FAF can be measured with already existing imaging technologies. For FAF, the excitation and filter settings of fluorescein angiography (FA) are used [14], whereas for NIA, the excitation and filter settings of indocyanine green angiography (ICGA) are used [1]. Indeed, NIA was first observed as a phenomenon in pre-injection images of ICGA [15].

FAF was introduced a decade earlier than NIA and imaging techniques for FA are much more widely available compared to those for ICGA. In addition, high-quality NIA images are more difficult to obtain due to the 60–100-fold lower NIA intensity compared to FAF. Standard ICGA fundus cameras and other devices for ICGA imaging do not provide sufficient sensitivity to record NIA images. The introduction of optical coherence tomography (OCT), which uses near-infrared wavelengths as well, has also affected NIA recording. NIA images can best be obtained with the confocal scanning laser ophthalmoscope HRA2 (Heidelberg Engineering, Heidelberg, Germany) including filters for ICGA. Spectralis OCT & ICGA Heidelberg Engineering devices introduced a different filter setting for ICGA to allow the passage of near-infrared illumination required for OCT. This different filter setting reduced the range of wavelengths available for NIA and reduced the quality of NIA images compared to the HRA recordings. Therefore, more experience is required to obtain NIA images with this setting. Special methods for obtaining NIA images with this setting have been reported [16,17]. Since ICGA is less widely available compared to FA, NIA can only be obtained with ICGA-capable Heidelberg Engineering devices, and experienced photographers are needed to obtain high-quality images, the use of NIA has been more limited compared to that of FAF. NIA was not included in recent standard recommendations for retinal imaging [18,19,20].

Degenerative, inflammatory, autoimmune and inherited disorders affecting the photoreceptor–RPE complex alter the interaction between photoreceptors and RPE cells, with subsequent changes in the distribution of melanin and lipofuscin. Changes in melanin and lipofuscin distribution vary regionally as well as during disease progression; therefore, NIA and FAF provide different insights into the pathophysiologic processes [21,22,23,24,25,26,27]. In various retinal disorders, and especially in inherited retinal dystrophies (IRDs), NIA can detect pathologic alterations in areas in which other established non-invasive retinal imaging methods like FAF or OCT still show normal findings [28,29].

NIA is a valuable tool for understanding pathophysiologic processes involving the RPE and provides important insights into the onset of disease processes, especially in IRD. So far, the current knowledge on NIA in IRD has been made available in multiple separate publications but has not been summarized in an overview. The purpose of the present review is to summarize NIA findings in IRD.

## 2. NIA—Near-Infrared Autofluorescence

In 2006, NIA was introduced as part of a standard protocol for retinal imaging in patients with IRD in our center, which specializes in the examination of IRD patients. Since then, 1963 IRD patients have been examined with NIA; follow-up NIA examinations were performed in 627 patients. Out of 1917 IRD patients, 1164 patients underwent molecular genetic analysis; in 715 of those, the genetic background was solved. The current review is based on this experience and a literature search of Pubmed, which was conducted using the terms “near-infrared autofluorescence”, “near-infrared fundus autofluorescence” as well as the abbreviations used in the literature: “NIA”, “NIR-FAF” and “NIR-RAFI”. The term RAFI (reduced-illumination autofluorescence imaging) was introduced by Cideciyan et al., though the settings were only reduced for FAF, but remained unchanged for NIA [30]. The final search update was performed on 1 September 2024.

Retinal imaging, including NIA, FAF, and spectral domain optical coherence tomography (OCT), was performed as described previously [31,32]. All images were obtained after medical dilatation of the pupil (which involved administering phenylephrine 2.5% and tropicamide 1%, achieving a minimal diameter of 5 mm). NIA and FAF were obtained with a confocal scanning laser ophthalmoscope (Heidelberg Retina Angiograph 2, Heidelberg Engineering, Heidelberg, Germany) using 30° and 50° lenses. The image resolution was 1023 × 1023 pixel. The maximum illumination of a 10 × 10° field of view was about 2 mW/cm^2^. OCT images were obtained with a spectral domain OCT (Spectralis OCT, Heidelberg Engineering, Heidelberg, Germany). Wide-field OCTs (50°) were added in 2016.

## 3. NIA Biomarkers in IRDs

### 3.1. NIA Alterations Indicative of RPE Pathology

**Figure 1:**
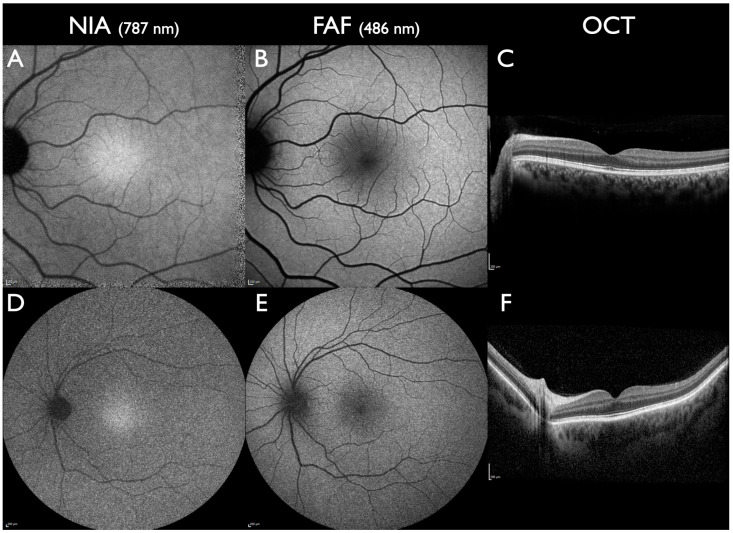
Normal NIA distribution. (**A**–**C**): 30° images. (**D**–**F**): 50° images. Corresponding to the distribution of melanin in RPE cells [10], the highest NIA intensity is located under the fovea, with a decline towards the parafovea and more peripheral homogenous intensity towards the periphery. The area of higher intensity varies between patients. Retinal vessels block NIA and appear dark, similar to the optic disc which contains no melanin. In contrast to FAF, NIA is not blocked by macular pigment and therefore facilitates better detection of foveal lesions. All scale bars indicate 200 µm.

Normal NIA distribution is displayed in Figure 1. Alterations of the normal NIA distribution present as either increased, reduced, or absent NIA intensity [1,31,33]. In contrast to FAF, an increase in NIA intensity is observed less frequently than reduced or absent NIA intensity. Increased NIA intensity can be due to an increase in melanin, e.g., in choroidal nevi or bilateral diffuse uveal melanocytic proliferation [21,34]. In progressive chorioretinal disorders, increased NIA intensity can occur due to increased melanogenesis, increased oxygenation of melanin, or increased formation of melanolipofuscin. An anterior displacement of melanin granules in the RPE cells could also contribute to an increased NIA intensity [4]. In the course of disease progression, areas with increased NIA intensity usually progress to areas with reduced NIA intensity.

Reduced NIA intensity can be associated with a loss of melanin in the RPE cells, a reduction in phagocytosis with reduced melanin activity, a different distribution of melanin granules in the RPE cells, the onset of RPE cell degeneration, or a relative blockade of the signal (e.g., exudation, blood). RPE cells underlying areas without active photoreceptors no longer contain measurable melanin [35,36,37]. In some acquired disorders, reduced NIA intensity can normalize during the healing process. This has been observed, e.g., in multiple evanescent white dot syndrome [38]. In IRDs, the NIA alterations are either permanent or progressive.

A nearly absent NIA intensity is associated with a loss of RPE cells, corresponding to window defects in fluorescein angiography, or severe blockage of the signal. In areas with RPE loss, a low NIA intensity originating in the choroid can be observed between the choroidal vessels.

Due to the normalization of the grayscale in NIA images, only changes in affected areas are measurable during the course of chorioretinal disorders. Variations in NIA intensity over time can only be evaluated qualitatively. Quantification of autofluorescence intensity in comparison with a standard probe has been investigated for FAF (qFAF), but not for NIA. However, even qFAF varies between different quantification methods and a generalized increase in intensity was only shown in *ABCA4*-associated IRDs [39,40]. A post-measurement mathematical approach for quantifying NIA intensity in non-normalized NIA images from normal probands and patients with chorioretinal disorders has been proposed by Paavo et al. [8,41]

### 3.2. NIA in Macular and Cone-Rod Dystrophies

#### 3.2.1. ABCA4-Associated IRDs (Stargardt Disease, Autosomal Recessive Cone-Rod Dystrophy)

**Figure 2:**
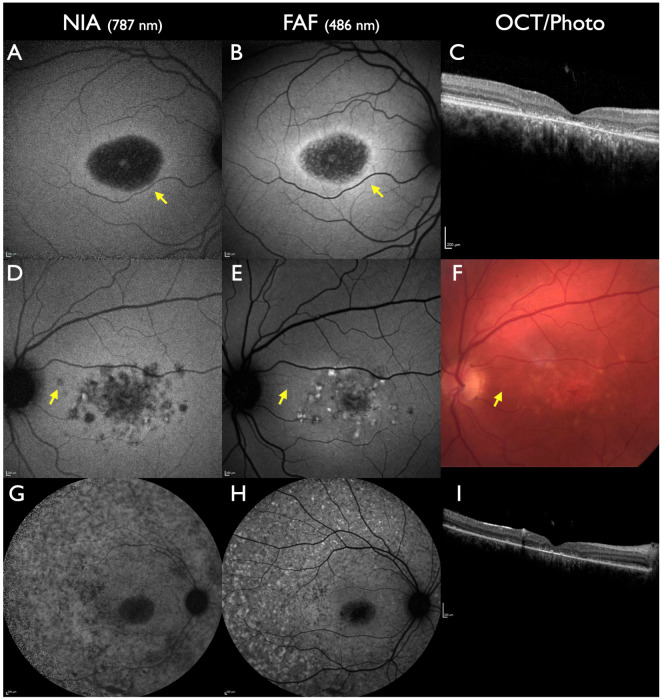
*ABCA4*-associated IRD. All patients had two pathogenic or likely pathogenic gene sequence variants in the *ABCA4* gene. (**A**–**C**): A 16-year-old male. Visual acuity 20/200 on the right eye, 20/400 on the left eye, and central scotomas. Severe loss of NIA and FAF intensity at the posterior pole with a small area of preserved subfoveal NIA and FAF. Towards the periphery, the ring of increased NIA intensity is slightly peripheral to the ring of increased FAF intensity (yellow arrow). (**D**–**F**): A 37-year-old male: visual acuity 20/200 on both eyes and central scotomas. Fleck-like areas of abnormal intensity are more extensive in NIA compared to FAF. A parapapillary fleck can be detected with NIA, but not with FAF or fundus photography (yellow arrows). (**G**–**I**): A 15-year-old patient with CRD. Visual acuity 20/200 on both eyes and central scotomas. Multiple fleck-like lesions in NIA and FAF, more reduced intensity in NIA compared to FAF. The area of preserved peripapillary RPE is smaller in NIA compared to FAF. All scale bars indicate 200 µm.

*ABCA4*-associated autosomal recessive IRDs present with huge phenotypic variability (Figure 2). This ranges from macular dystrophy confined to the posterior pole for the lifetime (i.e., Stargardt disease) to continuously peripheral progression involving the retina to the far periphery in late stages in cone-rod dystrophy (CRD). Animal studies in *ABCA4*-associated IRDs have demonstrated that an accumulation of lipofuscin granules in RPE cells develops at the beginning of the dystrophic process, followed by a subsequent loss of separate lipofuscin and melanin granules and an increase in melanolipofuscin granules [7,42]. These RPE alterations evolve prior to retinal functional loss in humans as well [30]. The ubiquitous increase in lipofuscin can only be measured with quantified FAF (qFAF) and not be demonstrated with normal FAF [40]. NIA depicts the irregular alterations of melanolipofuscin concentration in the RPE as the earliest sign.

The rate of disease progression is higher when measured with NIA compared to FAF [43]. During progression, NIA shows a faster reduction in intensity and less flecks with increased intensity compared to FAF. Serial NIA and FAF testing shows that the evolution of flecks with increased FAF intensity is preceded by alterations in NIA intensity in that location [33,44,45,46,47,48]. Consequently, the area of NIA lesions is larger compared to FAF [49,50,51]. In several patients, the area of reduced NIA intensity is larger than the loss of the ellipsoid zone in OCT and, therefore, is the earliest sign of RPE cell loss prior to photoreceptor decline [52,53,54,55]. A reduced subfoveal NIA intensity is usually associated with marked loss of visual acuity [56]. In contrast, in patients presenting a phenotype with preserved foveal structures (foveal sparing) good visual acuity corresponds to normal or near-normal subfoveal NIA intensity [57].

Peripapillary-preserved RPE is another biomarker that is frequently, but not exclusively, seen in *ABCA4*-associated CRD (Figure 2). This area can be detected with NIA and FAF but is smaller in NIA [58,59]. In severely progressed CRD, FAF can still detect fleck-like areas with relatively increased FAF intensity, whereas NIA intensity is markedly reduced or unmeasurable throughout the RPE.

#### 3.2.2. BEST1: Best Disease, Autosomal Recessive Bestrophinopathy and Autosomal Dominant Vitreoretinochoroidopathy

The earliest ophthalmoscopic sign of autosomal dominant Best disease associated with *BEST1* is a vitelliform lesion that is usually located centrally in the macula. During the individually variable course of progression, the vitelliform material is absorbed and, in late stages, an atrophic lesion develops, which is difficult to differentiate from atrophic lesions due to other atrophic disorders. In the initial stage, NIA intensity is increased within the vitelliform lesion [60,61]. During disease progression, the loss of NIA intensity precedes the loss of FAF intensity. Subfoveal preserved NIA intensity is associated with a better retinal sensitivity [62].

**Figure 3:**
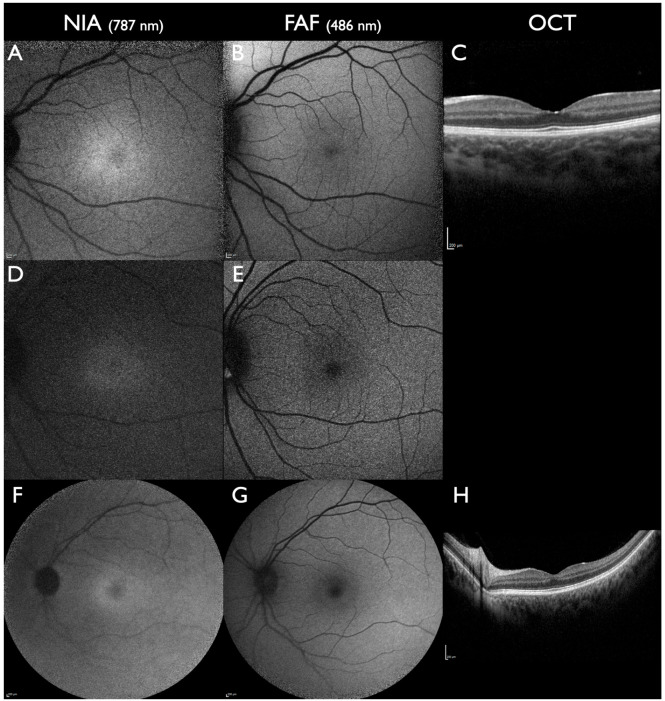
*BEST1*-associated macular dystrophy in subclinical stages. All patients had one pathogenic gene sequence variant in the *BEST1* gene (**A**–**C**): A 3-year-old female. Visual acuity 20/20; visual fields not tested due to age. Reduced subfoveal NIA intensity and normal FAF and OCT. (**D**,**E**): A 40-year-old female, aunt of the previous patient. Visual acuity 20/20; visual fields normal. Reduced subfoveal NIA intensity and normal FAF; OCT not performed. (**F**–**H**): A 44-year-old female from a different family. Visual acuity 20/20; visual fields normal. Reduced subfoveal NIA intensity and slightly increased parafoveal FAF intensity; normal OCT. All scale bars indicate 200 µm.

Best disease is known for incomplete penetrance. The earliest biomarker in a preclinical stage of Best disease is a subfoveal reduced NIA intensity [29]. In this stage, FAF, OCT, and ophthalmoscopy are completely normal (Figure 3). In non-manifesting carriers of Best disease, a subfoveal reduced NIA intensity remains the only detectable biomarker (Figure 3). In the next step, while ophthalmoscopy is still normal, initial alterations of the outer retinal layer in OCT and the foveal avascular zone in the deep retinal plexus in OCTA can develop [63].

In autosomal recessive bestrophinopathy (ARB), reduced subfoveal NIA intensity corresponded to choriocapillaris rarefication in OCTA and the development of non-vasogenic macular edema [64]. Similarly to *ABCA4*-associated IRD, peripapillary sparing can be documented with NIA and FAF in ARB. A reduction in the NIA intensity of the macular lesions corresponded to melanin loss in RPE cells in polarization sensitivity OCT (PS-OCT) [4]. Fleck-like increased NIA intensity corresponded to focally thickened RPE melanin associated with either stacked RPE cells or RPE cell dysmorphia [4].

Autosomal dominant vitreoretinochoroidopathy (ADVIRC) may be observed alongside non-progressive peripheral lesions, which are sometimes complicated by non-vasogenic macular edema. Some patients show a marked progression from the periphery to the posterior pole. The border between normal and affected retina is marked by increased NIA and FAF; the NIA appears slightly more centrally compared to FAF [65].

#### 3.2.3. NIA in Other Macular Dystrophies

Macular dystrophies present with a variation of phenotypes, some of them with a continuous overlap into CRD. An atypical phenotype of pseudoxanthoma elasticum was observed in one patient, and was associated with *ABCC6*. Patterned dystrophy-like lesions were associated with alterations of NIA and FAF intensity; the affected area was much larger in NIA compared to FAF [66].

**Figure 4:**
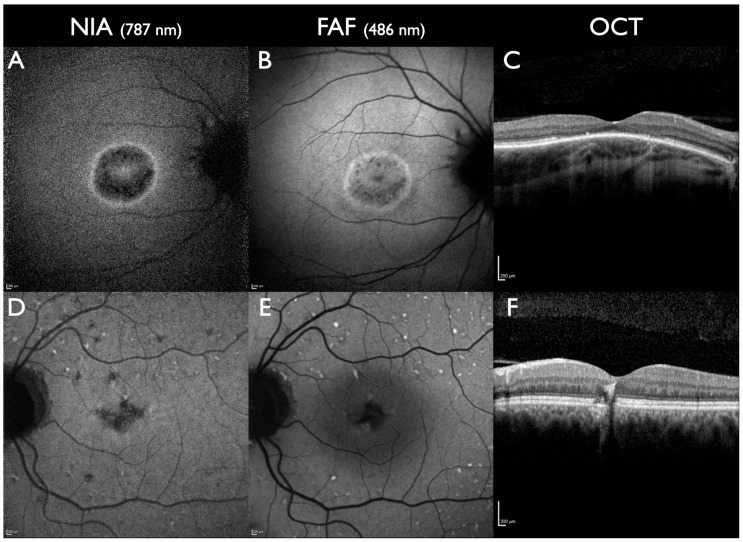
Cone-rod dystrophy. (**A**–**C**): A 43-year-old male. *RPGR*-associated CRD; one pathogenic gene sequence variant in the *RPGR* gene. Visual acuity 20/40 in the right eye and 20/25 in the left eye; paracentral scotomas. Central lesion with rings of increased NIA and FAF intensity, the ring is slightly larger in NIA. (**D**–**F**): 54-year-old male, *PRPH2* associated CRD, one pathogenic gene sequence variant in the *PRPH2* gene. Visual acuity 20/400 on the right eye, 20/40 on the left eye, central scotoma on the right eye, paracentral scotoma on the left eye. Reduced subfoveal NIA intensity is more extensive compared to FAF. Lesions with increased FAF intensity are predominantly located in larger areas with reduced NIA intensity. All scale bars indicate 200 µm.

In *PROM1*-associated macular dystrophy, NIA facilitated a better detection of foveal lesions compared to FAF [41]. Similarly, in central areolar choroidal dystrophy associated with *PRPH2*, NIA identified a larger lesion area compared to FAF. In addition, NIA and OCTA identified the earliest signs of disease onset in RPE and choriocapillaris [67]. In another study, macular dystrophy associated with *PRPH2* foveal and parafoveal lesions was more widespread in NIA compared to FAF [68] (Figure 4).

#### 3.2.4. NIA in Cone-Rod Dystrophies

The heterogeneous group of cone-rod dystrophies (CRDs) is associated with pathogenic variants in more than 60 genes. The clinical phenotype can be variable between different genes, but also within one family with the same genetic background. The funduscopic findings do not allow differentiation between different genetic associations or non-syndromic or syndromic CRDs.

CRD can present with a lesion at the posterior pole surrounded by rings of increased NIA and FAF intensity that are opposite to retinitis pigmentosa (RP) [69]. Peripheral to that ring, NIA and FAF can appear normal or present fleck-like alterations (Figure 4). Other forms of CRD present without rings but with multiple fleck-like lesions from the posterior towards the periphery in NIA and FAF (Figure 4). In CRD, loss of NIA intensity is more severe and widespread compared to FAF [68,69,70,71,72,73].

A foveal cavitation—a central area with absence of the outer retinal layers—is not uncommon in CRD and achromatopsia. Within the area of cavitation, NIA and FAF intensity are reduced. The area of reduced NIA intensity is larger compared to the area of reduced FAF intensity and larger than the cavitation determined by OCT [74].

### 3.3. NIA in Retinitis Pigmentosa

The heterogeneous group of retinitis pigmentosa (RP) is associated with pathogenic variants in more than 130 genes. It mostly presents as non-syndromic RP, but RP is a frequent phenotype in syndromes involving the retina. There is a huge variability in onset and disease progression associated with different genes. In addition, severity of the phenotype can also be variable between members of the same family. The funduscopic findings do not allow differentiation between different genetic associations or non-syndromic or syndromic RP.

**Figure 5:**
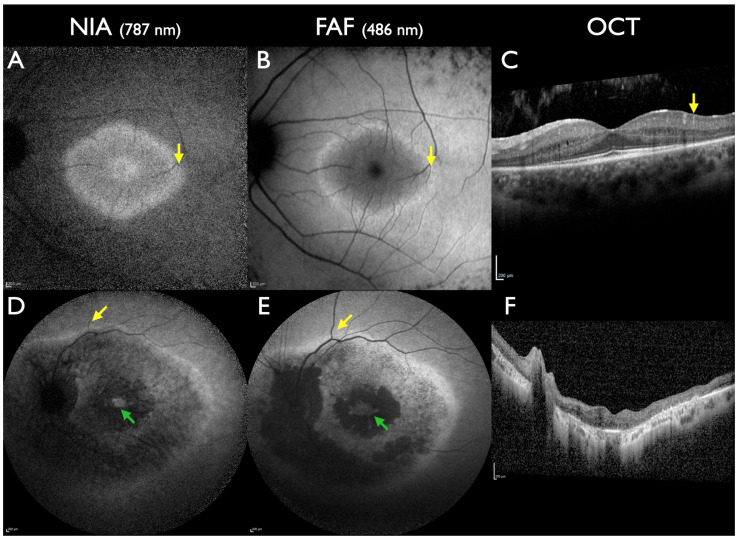
Retinitis pigmentosa. (**A**–**C**): A 35-year-old female. *PRPF6*-associated autosomal dominant RP; one likely pathogenic gene sequence variant in the *PRPF6* gene. Visual acuity 20/20; concentric constriction of visual field. Pericentral ring of increased NIA and FAF intensity and marked adjacent peripheral reduction in NIA, but not FAF intensity. The same vessel is indicated by yellow arrows in NIA, FAF, and OCT. The ring of increased intensity is slightly smaller in NIA. The ring in NIA corresponds to the end of the EZ line on OCT. (**D**–**F**): A 54-year-old female. *NPHP1*-associated autosomal recessive syndromic RP; one homozygous *NPHP1* gene sequence variant. Visual acuity in one the right eye in response to hand movement; in the left eye, 20/400 ring scotomas. Mid-peripheral ring of increased NIA and FAF intensity; the ring is slightly more peripheral compared to FAF (yellow arrows). The central area of preserved NIA intensity is smaller compared to the preserved FAF intensity (green arrows). All scale bars indicate 200 µm.

A characteristic biomarker of RP is a ring of increased intensity in NIA and FAF surrounding the area of preserved photoreceptor (Figure 5). The ring indicates the border of disease progression and correlates with the remaining visual field [31,75,76,77,78,79,80]. Compared to FAF, the ring in NIA is slightly smaller, indicating that RPE alterations are first observed with NIA [81,82]. Slightly faster progression was observed in NIA [82]. The area of preserved NIA correlates with the preserved ellipsoid zone in OCT. Directly adjacent to this ring, towards the periphery, NIA intensity markedly drops, whereas FAF intensity appears quite normal [31,83]. This difference correlates with the loss of photoreceptors and the loss of melanin in RPE cells when functioning photoreceptors are absent, which is indicated by loss of NIA intensity [35,36,37,84,85]. The preserved RPE in that area still contains lipofuscin, as demonstrated by preserved FAF. In more peripheral areas with loss of RPE, NIA and FAF are both markedly reduced.

Areas with preserved NIA also correspond with vascular structures. In areas with absent NIA intensity, OCTA demonstrated marked alterations of the retinal and choroidal vasculature [86].

### 3.4. NIA in Choroideremia

**Figure 6:**
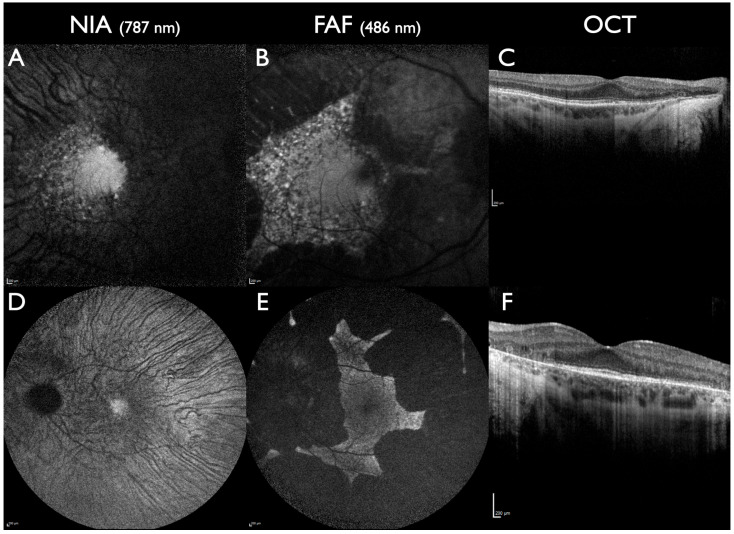
Choroideremia. Both patients had one pathogenic gene sequence variant in the *CHM* gene. (**A**–**C**): A 56-year-old male. Visual acuity 20/25 in both eyes; severely constricted visual fields. The area of preserved NIA intensity is smaller than the area of preserved FAF intensity and the preserved ellipsoid zone in OCT. (**D**–**F**): A 23-year-old male. Visual acuity 20/20 in both eyes; severely constricted visual fields. The area of preserved NIA intensity is smaller than the area of preserved normal FAF intensity, much smaller than the area of mottled FAF intensity, and smaller than the preserved ellipsoid zone in OCT. NIA from choroidal melanin is detectable between large choroidal vessels. All scale bars indicate 200 µm.

X-linked choroideremia is associated with *CHM*. In males, loss of RPE and photoreceptors starts in the periphery and continuously progresses towards the fovea. Reduction in NIA intensity is more widespread than in FAF, indicating that initial RPE alterations can be detected with NIA, even in young children [87]. Areas of measurable FAF are usually much larger than the areas of NIA (Figure 6). However, FAF presents with two different aspects: central areas of preserved FAF and adjacent areas of mottled FAF with fleck-like FAF intensity reduction [88]. NIA intensity is severely reduced in areas with mottled FAF [89,90] and the area of preserved NIA is usually smaller than the area of preserved FAF. In some patients, the area of preserved NIA is smaller than the preserved ellipsoid zone in OCT [90]. Subfoveal preserved NIA correlates with better visual acuity [89,90]. This could be a helpful biomarker for follow-up of the course of the disease, as well as for selection of patients for therapeutic studies.

Female carriers present with variable clinical signs, ranging from absence of symptoms to severe functional loss similar to that experienced by males. Independent of the symptoms, ophthalmoscopic visible lesions are present in all carriers, but more lesions can be detected with FAF and even more can be detected with NIA [89,90,91].

Fundus, NIA and FAF alterations similar to choroideremia were observed in a patient with Boucher–Neuhauser syndrome associated with PNPLA6 [92].

## 4. NIA Imaging in IRD: Summary and Future Perspectives

The present review summarizes the experience of NIA imaging in IRD since its introduction in 2006 (Table 1). Alterations of FAF and NIA are present in nearly all IRD phenotypes and autofluorescence imaging allows a detailed analysis of RPE alterations. NIA intensity alterations usually precede FAF alterations as the earliest sign of RPE abnormalities in IRDs. These findings are important for understanding the pathophysiologic processes in different IRDs. Though NIA is not required as a diagnostic tool for every IRD in clinical practice, NIA can serve as a biomarker for earliest changes, as well as a potential criterium for patient selection for future clinical trials as well as in animal models. In families with autosomal dominant Best disease, only NIA can detect subclinical stages even in young children. An additional advantage of using NIA for the examination of children is that it causes less discomfort due to the absence of glare compared to FAF.

Recently, different techniques have been described for measuring near-infrared autofluorescence. Polarization-sensitive OCTs provide entropy measurements of RPE melanin. Initial studies have been performed in healthy eyes, as well as in patients with retinitis pigmentosa and Best disease, and demonstrate a good correlation between NIA and PS-OCT imaging [4,80,93]. Excitation with a shorter wavelength of 636 nm has been used in an adaptive optics scanning laser ophthalmoscope [94], but few patients have been examined so far. Near-infrared adaptive optics fluorescence lifetime imaging ophthalmoscopy (NIR-AFLIO) showed regional differences in healthy subjects with longer lifetimes towards the periphery [95]. NIR-AFLIO showed prolonged lifetimes in areas with early AMD. This technique might be a possible instrument for in vivo monitoring of early disease processes.

## Figures and Tables

**Table 1:** Features of NIA, FAF and OCT imaging in IRDs.

Disorder	NIA	FAF	OCT	Comparison
*ABCA4*-associated disorders	-Variable: multiple fleck-like lesions or central lesions surrounded by a ring of increased intensity -Marked reduction in intensity in progressed stages -Peripapillary sparing -Subfoveal intensity loss associated with reduced VA [56,57]	-Variable: multiple fleck-like lesions or central lesions surrounded by a ring of increased intensity -Peripapillary sparing	-Subretinal deposits -Loss of outer retinal layers (EZ line) and RPE	-NIA alterations precede FAF alterations: -Affected area with NIA larger compared to FAF and OCT [49,50,51] -Area of reduced intensity larger than EZ loss in OCT [52,53,54,55]
Preclinical Best macular dystrophy	Subfoveal reduced intensity [29,63]	Normal	Normal	Only detectable by NIA
Best macular dystrophy	-Lesions with increased or reduced intensity -Areas of reduced intensity increasing during progression -Subfoveal preserved NIA intensity associated with better VA [62]	-Lesions with increased or reduced intensity -Areas of reduced intensity increasing during progression	-Subretinal fluid and/or subretinal material -Atrophy of outer retinal layers (EZ line) and RPE in late stages	Reduced NIA intensity precedes reduced FAF intensity [62]
Autosomal recessive Bestrophinopathy	-Areas of variably increased or reduced intensity -Peripapillary sparing	-Areas of variably increased or reduced intensity -Peripapillary sparing	-Subretinal material, subretinal fluid -Atrophy of outer retinal layers (EZ line) and RPE in late stages	Subfoveal reduced NIA intensity corresponds to vascular rarefication and CME [64]
Macular dystrophies	Variable: multiple fleck-like lesions or central lesions surrounded by a ring of increased intensity	Variable: multiple fleck-like lesions or central lesions surrounded by a ring of increased intensity	-Subretinal deposits -Loss of retinal outer retinal layers (EZ line) and RPE	-Lesion area larger compared to FAF in MD associated with *ABCC6*, *PROM1*, and *PRPH2* [41,66,68] -NIA intensity reduction as earliest sign in *PRPH2* compared to FAF/OCT [67]
Cone-rod dystrophies	-Variable: multiple fleck-like lesions or pericentral ring of increased intensity with centrally reduced intensity -Reduced intensity in foveal cavitation	Variable: multiple fleck-like lesions or pericentral ring of increased intensity with centrally reduced intensity	Variable: subretinal deposits, loss of outer retinal layers (EZ line) and RPE, foveal cavitation in some cases	-Loss of NIA intensity more wide-spread compared to FAF [68,69,70,71,72,73] -In foveal cavitation, NIA intensity loss is larger compared to OCT EZ line loss [74]
Retinitis pigmentosa	-Pericentral ring of increased intensity corresponding to the end of the EZ line in OCT -Marked intensity loss peripherally to the ring in areas with preserved or absent RPE	-Pericentral ring of increased intensity corresponding to the end of the EZ line in OCT -Preserved intensity adjacent to the ring and marked loss further peripherally	Variable: centrally preserved or reduced outer retinal layers (EZ line), towards the periphery loss of outer retinal layers and RPE, CME in about 50% of cases	-Ring of increased intensity in NIA more centrally compared to FAF [81,82] -Absent NIA intensity corresponds to vascular alterations in OCTA [86]
Choroideremia	-Early-onset wide-spread reduction in intensity -Small areas of preserved intensity correlate to unspeckled area of FAF intensity	-Multiple irregular areas of reduced intensity, -Central area of preserved intensity surrounded by an area with speckled loss of intensity	Variable: centrally preserved or reduced outer retinal layers (EZ line) towards the periphery; increasing areas of loss of outer retinal layers and RPE	-Area of preserved NIA smaller compared to FAF [90] -Subfoveal preserved NIA correlates with better VA [89,90]

## Data Availability

The data presented in this study are available on request from the corresponding author due to legal restriction.
